# Integrating Eye Tracking and Self-Report for Sensory–Emotion Evaluation of Red Bean and Coix Seed Water: A Complementary Validation Framework

**DOI:** 10.3390/foods15152628

**Published:** 2026-07-27

**Authors:** Haoqun Zhang, Yue Zhao, Yuli Hao, Xiaojing Leng, Yizhen Huang

**Affiliations:** 1 College of Food Science and Nutritional Engineering, China Agricultural University, Beijing 100083, China; 2 Faculty of Health and Wellness, City University of Macau, Macau SAR, China

**Keywords:** eye tracking, sensory–emotion evaluation, self-report questionnaire, area of interest (AOI), implicit and explicit measures, consumer preference

## Abstract

To examine whether gaze behavior on the rating scale can complement conventional self-report, twenty-four volunteers evaluated three red bean and coix seed water beverages on sensory attributes, EsSense-based emotional terms, and preference, while their fixations on the response scale were recorded using a synchronous area-of-interest design. Two indices were extracted: the questionnaire rating (QR), reflecting the consciously selected score, and the eye tracking-derived rating (ETR), defined as the longest-fixated score option. QR and ETR were strongly correlated (ICC: 0.88–0.89) and produced an identical preference ranking (R1 > R2 > R3), yet the wide limits of agreement indicated that they are not interchangeable at the individual level. Across the sensory–preference, sensory–emotion, and emotion–preference layers, QR consistently yielded stronger correlations than ETR. Warmth and sweetness were positively associated with positive emotions and liking, and “Good-natured” was the most stable emotional correlate of liking. Gaze behavior thus provides a process-oriented complement to, rather than a replacement for, self-report.

## 1. Introduction

Accurately capturing consumers’ perceptual and emotional experiences of food has long been a central goal of sensory evaluation, yet this goal has persistently been constrained by two categories of methodological obstacles [1].

The first obstacle stems from the reliance on verbal report. Conventional self-report is susceptible to memory bias, anchoring effects, and social desirability, and many subconscious cognitive and emotional responses cannot be fully articulated in words [2,3]. Although standardized tools such as check-all-that-apply (CATA) and rate-all-that-apply (RATA) have improved evaluation efficiency to some extent [4,5], they have not fundamentally overcome this limitation. Köster noted that the dimensions along which humans perceive food far exceed what language can describe [6]; Ares et al. found that consumers tend to select terms that are easy to name, giving rise to a “term accessibility” bias [4]; and the “verbal overshadowing” effect reported by Melcher and Schooler further indicates that verbalization itself may interfere with, or even distort, the original sensory memory [7]. Even with the introduction of emotion measurement tools such as the EsSense Profile™ [8,9] and EmoSemio [10], these approaches essentially address the same problem with a larger vocabulary, and the dependence on verbal mediation remains.

The second obstacle arises from implicit measures that attempt to bypass language. Researchers have introduced neuroimaging techniques—electroencephalography (EEG), functional near-infrared spectroscopy (fNIRS), and functional magnetic resonance imaging (fMRI)—as well as peripheral physiological indices such as facial electromyography (EMG) and eye tracking (ET) to compensate for the shortcomings of explicit measures [11]. However, integrating such data with self-reporting poses multiple difficulties: continuous physiological signals and ordinal ratings are difficult to match in temporal scale and measurement unit, and are therefore not readily cross-validated [12]; the correspondence between physiological responses and subjective emotion is unstable, as the same physiological pattern may map onto different emotional states [13]; and differences in sampling frequency, noise characteristics, and individual variability across devices further compound the complexity of cross-modal alignment [14]. Consequently, although implicit measures provide rich information, how to achieve their effective fusion with explicit data remains a current methodological challenge.

Among these implicit measures, eye tracking has attracted considerable attention for its non-invasiveness and its capacity to record the spatiotemporal distribution of visual attention in real time. Supported by a body of theoretical and empirical work—the “eye–mind hypothesis” (the fixated point being the object of cognitive processing) [15], the stable association between gaze behavior and decision making [16], the “gaze cascade effect” (fixation duration shifting systematically toward the eventually chosen option [17]; and the attentional drift diffusion model (aDDM)—eye tracking has come to be regarded as an effective tool for revealing implicit psychological processes [18,19]. Nevertheless, existing studies have largely been confined to visual stimulus contexts, have rarely examined gaze behavior directed at the rating scale itself, and have not yet addressed the integration of multimodal stimuli such as olfaction and taste in food sensory evaluation.

Our previous work has demonstrated the predictive validity of eye tracking for preference [20,21], but has not yet achieved its synchronous acquisition and complementary validation with self-report data. To address this gap, the present study constructs an implicit–explicit complementary validation framework by synchronously recording participants’ gaze behavior on the rating scale together with their questionnaire ratings, to examine whether gaze behavior directed at the scale can provide information that complements conventional self-report. Red bean and coix seed water was selected as the sample: as a widely known traditional medicinal and edible beverage particularly favored by younger consumers, it allows the consistency and divergence between eye tracking and self-report data in representing the “sensory–emotion–preference” association structure to be systematically compared while holding the basic beverage type constant, thereby testing the feasibility of the proposed approach.

## 2. Materials and Methods

### 2.1. Panelists

Twenty-four postgraduate students (11 men, 13 women) from the College of Food Science and Nutritional Engineering were recruited. All panelists had normal sensory function confirmed by standard screening tests and prior professional training in food sensory evaluation. Written informed consent was obtained from all panelists. The protocol was approved by the Ethics Committee of China Agricultural University (approval no. CAUHR-20260415).

### 2.2. Sensory Evaluation

**Figure 1:**
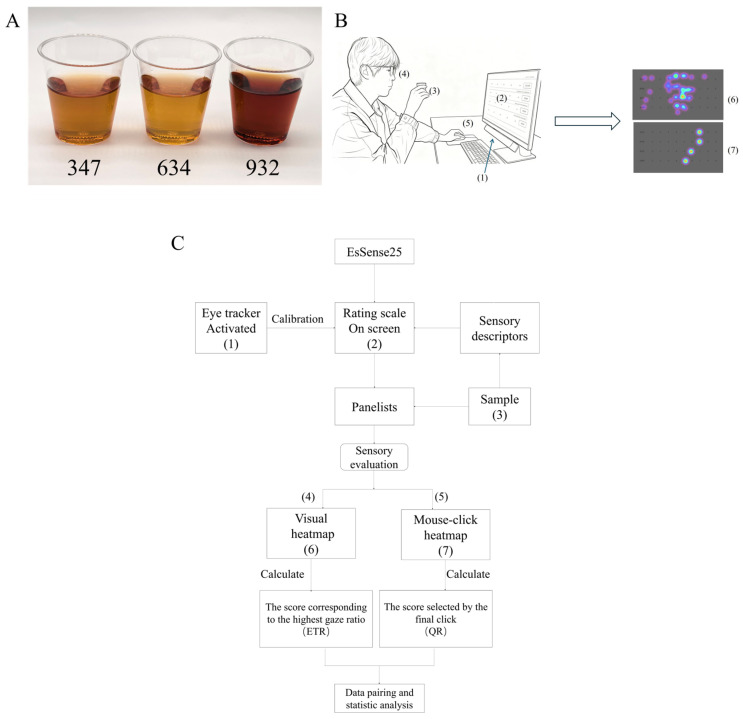
Sample photographs and experimental procedures. Note: (**A**) Sample photographs; (**B**) Schematic diagram of the experimental procedure. Note: for experimental procedure: (1) Eye tracker; (2) Electronic rating scale; (3) Beverage sample; (4) Participant’s pupil; (5) Click to complete rating; (6) Visual heatmap from participant’s gaze; (7) Click heatmap from participant’s clicks. The intensity order in the heatmaps is always red (highest), yellow, green, and blue (lowest); (**C**) Flow chart of the Experimental Protocol. Notes: The numbers in (**C**) correspond to the numbers in (**B**).

Three commercial red bean and coix seed water beverages were purchased from a local supermarket and labeled with random three-digit codes. Their ingredients and color characteristics are summarized in Table 1, and photographs are shown in Figure 1. All samples were stored at 4 °C and equilibrated to room temperature (22 ± 2 °C) before testing.

### 2.3. Attributes

A sensory lexicon was constructed across preference, visual, olfactory, gustatory, and tactile dimensions based on the literature, an industry term bank, and attributes elicited from panelists (Table 2, 9-point scale [22]). Emotional terms were assessed using the internationally established EsSense25 questionnaire (Table 3) [8], with terms classified by high/low arousal and high/low valence [23].

### 2.4. Instruments

A desktop eye tracker (Eyeso EC80, Xintuo Yingqi Technology Co., Ltd., Beijing, China) with accompanying software was used. Key specifications: sampling rate 150 Hz, accuracy 0.5°, precision 0.08°; viewing distance 50–90 cm; head movement range 25 × 20 × 40 cm; stimuli presented on a 23-inch LCD monitor (1920 × 1080; model 4P2U, TPV Technology (Fujian) Co., Ltd., Fuqing, China). The software automatically generated an electronic rating form in which each descriptor and its response options were defined as separate AOIs, and fixation durations were recorded.

Panelists rated each sample on a 9-point scale (1 = strongly disagree, 9 = strongly agree). Mouse clicks were recorded to generate the questionnaire rating (QR), while the eye tracking-derived rating (ETR) was generated synchronously and defined as the score of the AOI with the longest fixation duration. It should be noted that the longest fixation may reflect either preference or cognitive hesitation; this definition is adopted here as an exploratory operationalization (see Section 4.4). Panelists rinsed their mouths after each sample. Both QR and ETR are ordinal scores generated in pairs within the same task, achieving natural alignment in temporal sequence and information unit.

### 2.5. Experimental Protocol

The experimental procedure is illustrated in Figure 1B. The eye tracker was first activated (1), and gaze calibration was completed for each participant; an electronic rating scale was then presented on the screen (2). Participants evaluated the beverage samples (3) in sequence, during which the eye tracker collected gaze data by recording participants’ pupil (4) movements, while participants entered their ratings via mouse clicks (5). Visual heatmaps (6) were generated from the gaze trajectories, and mouse-click heatmaps (7) were generated from the clicking behavior.

Each panelist completed three independent replicate sessions for every sample within one week (≥24 h apart). Sample presentation order was balanced across panelists using a Williams design. Because the test–retest ICC(2,3) was below 0.75 for most attributes, averaging was deemed inappropriate [24]; the median was therefore used as each panelist’s individual score.

### 2.6. Statistical Analyses

Agreement between the two rating methods was assessed using Bland–Altman analysis, ICC (2,1), and Spearman’s rank correlation. Differences between methods and among samples were tested using Wilcoxon signed-rank tests (FDR-corrected) and Friedman tests, respectively. Sensory–emotion–preference associations were examined with repeated-measures correlation (Rmcorr, r_m_), with FDR correction applied to the resulting *p*-values; dispersion was compared using the median absolute deviation (MAD) and its confidence interval [25]. Distribution normality was evaluated with Pearson’s skewness coefficient, and the Kolmogorov–Smirnov test (K-S test) was used to compare the overall shape of the correlation coefficient distributions between QR and ETR. All statistical analyses were performed using IBM SPSS Statistics version 21.0 (IBM Corp., Armonk, NY, USA), and figures were generated using GraphPad Prism version 9.0.0 (GraphPad Software, San Diego, CA, USA).

## 3. Results

### 3.1. Agreement and Reliability Between ETR and QR

**Figure 2:**
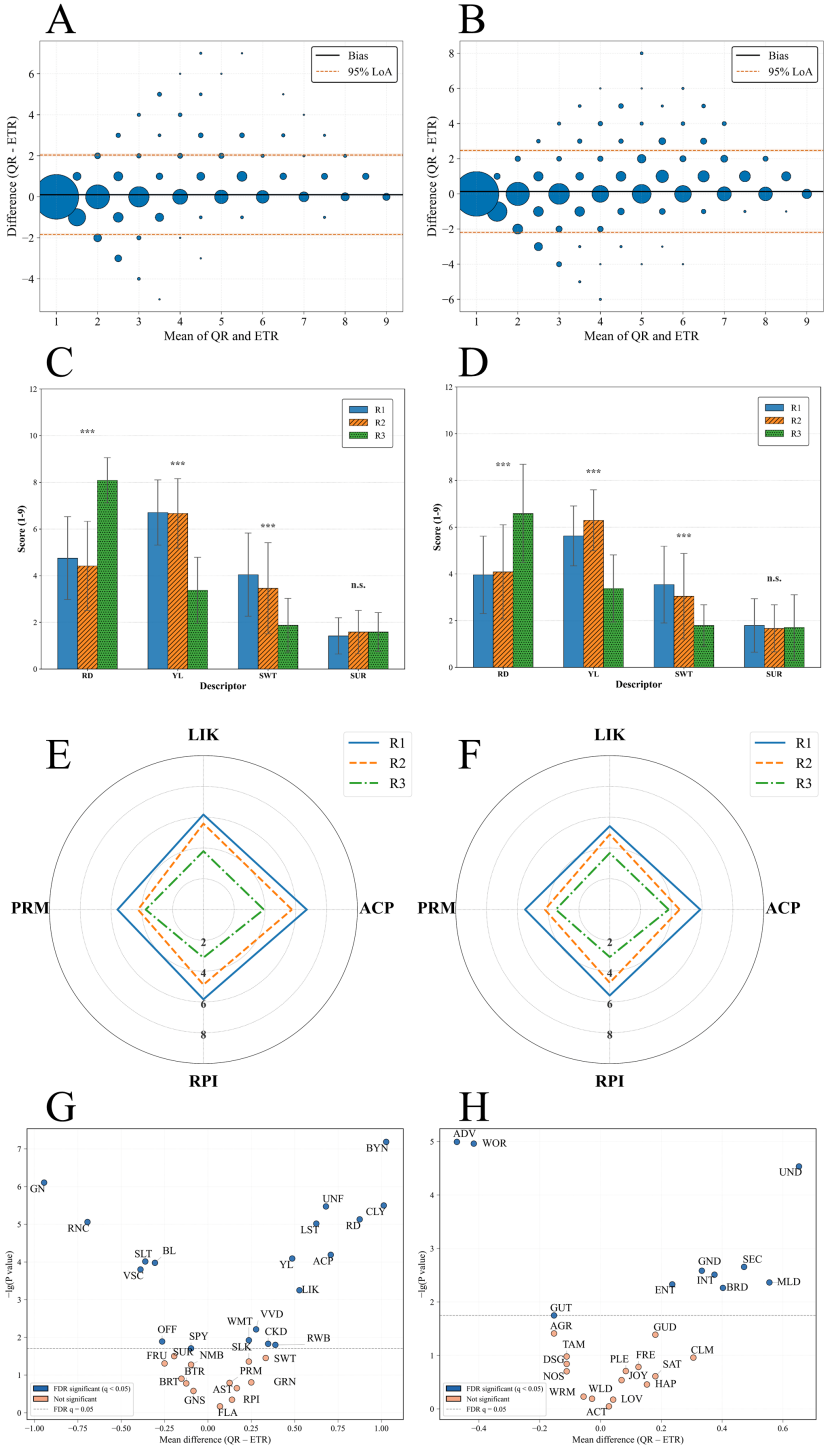
Comparative Reliability Analysis of Eye Tracking and Self-Report Measures in Sensory Evaluation. Note: (**A**,**B**) Consistency analysis between QR and ETR; (**A**) EsSense25; (**B**) Sensory–preference. The solid line represents the mean of the differences (ETR-QR), and the dashed lines represent the 95% limits of agreement (95% LoA = bias ± 1.96 SD). For EsSense25, bias = 0.10, 95% LoA = [−1.83, 2.04]; for the sensory–preference questionnaire, bias = 0.14, 95% LoA = [−2.19, 2.47]. The area size of each bubble reflects the relative frequency of observations at that coordinate (based on the overall analysis across all samples, panelists, and attributes). (**C**,**D**) Comparison of sensory attributes for red bean and coix seed water beverage; Note: (**C**) = QR, (**D**) = ETR; *** means *p* < 0.001 in the Friedman test. (**E**,**F**) Radar plot of mean ratings for three samples on LIK (liking), ACP (acceptance), RPI (re-purchase intention), and PRM (premiumness), (**E**) = QR, (**F**) = ETR. (**G**,**H**) Comparison of QR and ETR ratings for each descriptor using the Wilcoxon signed-rank test. Note: (**G**) EsSense25; (**H**) Sensory–preference. The dashed line marks the *p* = 0.05 significance threshold. Blue points show significant inter-method differences after FDR correction, and red points represent non-significant outcomes after FDR correction.

Agreement and reliability between the questionnaire-based ratings (QR) and the eye tracking-derived ratings (ETR) were assessed across all samples, panelists, and attributes using Bland–Altman analysis, intraclass correlation coefficients (ICC), and Spearman’s rank correlation (Figure 2A,B). ETR and QR achieved acceptable agreement and good reliability at the aggregate level, while the comparatively wide limits of agreement suggest that the two measures cannot be regarded as interchangeable at the individual level. The mean bias between the two methods was close to zero for both instruments (EsSense25: bias = 0.10; sensory–preference questionnaire: bias = 0.14), indicating the absence of any systematic overall offset. However, the 95% limits of agreement (LoA) were relatively wide, spanning [−1.83, 2.04] for EsSense25 and [−2.19, 2.47] for the sensory–preference questionnaire. Expressed as a proportion of the nine-point scale range, the standard deviation of the differences corresponded to 12.4% (EsSense25) and 14.9% (sensory–preference), both falling within the “good” (10%) to “acceptable”(25%) interval, respectively. Observations of perfect QR–ETR concordance were concentrated in the lower score region. Reliability was good for both instruments, with ICC(2,1) values of 0.88–0.89 and Spearman coefficients of 0.86–0.88 [24].

### 3.2. Discrimination of Samples by Perception and Preference

The discriminative capacity of the two methods was first examined using four readily perceivable anchor attributes (red, yellow, sweet, and sour; Figure 2C,D). ETR reproduced the discriminative conclusions of the explicit ratings. The two methods yielded highly consistent rating patterns. For the three anchor attributes expected to differentiate the samples based on their appearance and formulation—red, yellow, and sweet—both QR and ETR discriminated significantly among the three beverages (Friedman test, all *p* < 0.001), whereas for sour, which did not differ across formulations, neither method detected a significant difference (*p* > 0.05). The radar profiles of the four preference dimensions—liking (LIK), acceptability (ACP), repurchase intention (RPI), and perceived premiumness (PRM)—are presented in Figure 2E,F. The preference ranking was R1 > R2 > R3 across all four dimensions, with significant between-sample differences for every dimension (Friedman test, *p* <0.05); QR and ETR produced identical orderings.

### 3.3. Attributes-Level Differences Between the Two Methods

To identify the specific attributes on which ETR and QR diverged systematically, Wilcoxon signed-rank tests with false discovery rate (FDR) correction (Benjamini–Hochberg procedure, q < 0.05) were applied to each descriptor (Figure 2G,H). Among the attributes with a mean above five on the nine-point scale, significant ETR-QR differences emerged primarily for the visual attributes (uniformity, gloss, clarity, and red) and the olfactory attribute (bean aroma), whereas the remaining attributes did not differ significantly. For the preference dimensions, the two ratings differed significantly for acceptability and liking but not for repurchase intention or perceived premiumness. For the emotional dimension, significant differences were observed for the high-arousal/high-valence term “Adventurous” and for the low-arousal/low-valence terms “Guilty” and “Worried”, with valence and arousal defined according to Russell’s circumplex model of affect [23]. Overall, visual/appearance attributes tended to be rated slightly higher under QR, whereas certain emotional attributes tended to be higher under ETR, suggesting that the two indices emphasize different aspects of information across attribute types.

### 3.4. Sensory–Preference Associations

**Figure 3:**
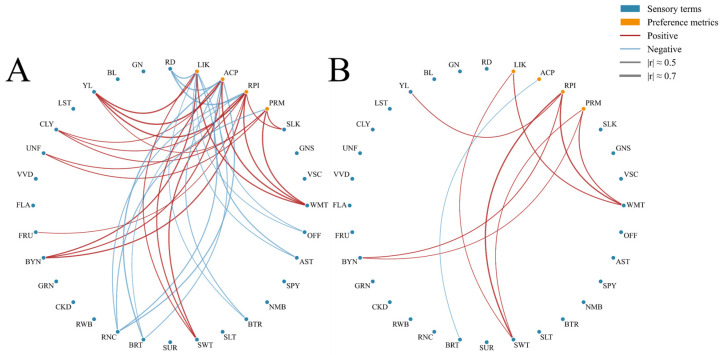
Correlation Analysis Between Sensory and Preference Descriptors Based on Repeated-Measures Correlation Levels. Note: Statistical significance was corrected using the FDR method (Benjamini–Hochberg, q < 0.05). (**A**) QR correlation chord diagram: significant sensory–preference descriptor pairs with |r| > 0.4 (based on all samples and panelists); (**B**) ETR correlation chord diagram: significant sensory–preference descriptor pairs with |r| > 0.4 (based on all samples and panelists); Red lines: positive correlations; blue lines: negative correlations; line width reflects correlation strength (wider line indicates larger |r|).

Repeated-measures correlations between sensory and preference attributes are summarized in Figure 3. The QR- and ETR-based chord diagrams (Figure 3A,B; FDR-corrected, q < 0.05) display descriptor pairs with |r| > 0.4. Summary statistics for the distribution of correlation coefficients from both methods are presented in Table 4. The dispersion of the correlation coefficients was significantly greater for QR than for ETR, with a 95% CI [1.26, 2.01] for the MAD ratio (QR/ETR), whose lower bound exceeded 1.0, indicating a statistically significant difference in dispersion between the two methods [25]. Correlation strength was likewise significantly higher for QR than for ETR (paired Wilcoxon signed-rank test, N = 112, *p* < 0.001). Both distributions showed small, comparable skewness (QR = −0.028, ETR = +0.131; Bootstrap sign test, *p* = 0.804). Kolmogorov–Smirnov tests further confirmed that the overall distribution shapes differed significantly between the two methods (*p* = 0.039). Among the sensory attributes significantly correlated with more than three preference terms, QR identified visual (yellow, red, clarity, and uniformity), olfactory (bean aroma), gustatory (sweet), and tactile (warmth, smoothness) attributes, whereas ETR identified only warmth and sweet. The drivers common to both methods—warmth (QR: r_m_ = 0.57–0.60; ETR: 0.28–0.59) and sweet (QR: 0.39–0.57; ETR: 0.38–0.61)—were all positively associated with preference.

### 3.5. Sensory-Emotion Correlation

**Figure 4:**
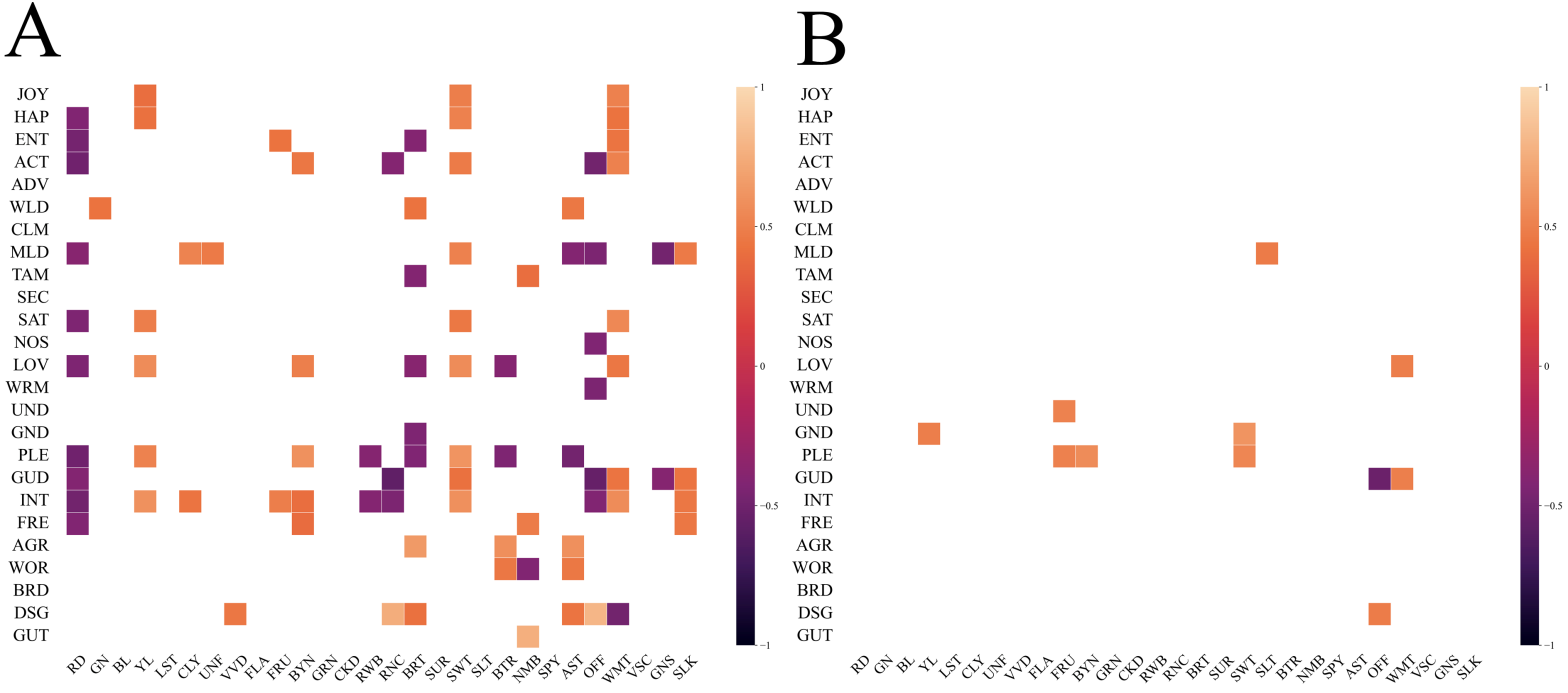
Association Analysis Between Sensory and Emotion Terms Based on Repeated-Measures Correlation Levels. Note: Statistical significance was corrected using the FDR method (Benjamini–Hochberg, q < 0.05); (**A**) QR correlation heatmap (based on all samples and panelists); (**B**) ETR correlation heatmap (based on all samples and panelists).

Repeated measures correlations between sensory and emotional attributes are shown in Figure 4, comprising QR and ETR correlation heatmaps (Figure 4A,B; FDR-corrected, q < 0.05). Summary statistics for the distribution of correlation coefficients from both methods are presented in Table 5. The two methods still showed significantly different dispersion (QR: MAD = 0.184; ETR: MAD = 0.127), with a 95% CI [1.26, 1.61] for the MAD ratio (QR/ETR), whose lower bound exceeded 1.0, indicating significantly greater dispersion for QR than for ETR [25]. Correlation strength remained significantly higher for QR than for ETR (paired Wilcoxon signed-rank test, N = 700, *p* < 0.001). Both distributions were positively skewed (QR = +0.196, ETR = +0.258; Bootstrap sign test, *p* = 0.466). Kolmogorov–Smirnov tests further confirmed that the overall distribution shapes differed significantly between the two methods (*p* < 0.001). Across both methods, the following sensory–emotion associations were consistently significant after FDR correction (q < 0.05): warmth was positively associated with “Loving” and “Good-natured”; bean aroma and sweet were positively associated with “Pleasant”; off-flavor was negatively associated with “Good-natured” and positively associated with “Disgusted”.

### 3.6. Emotion-Preference Associations

**Figure 5:**
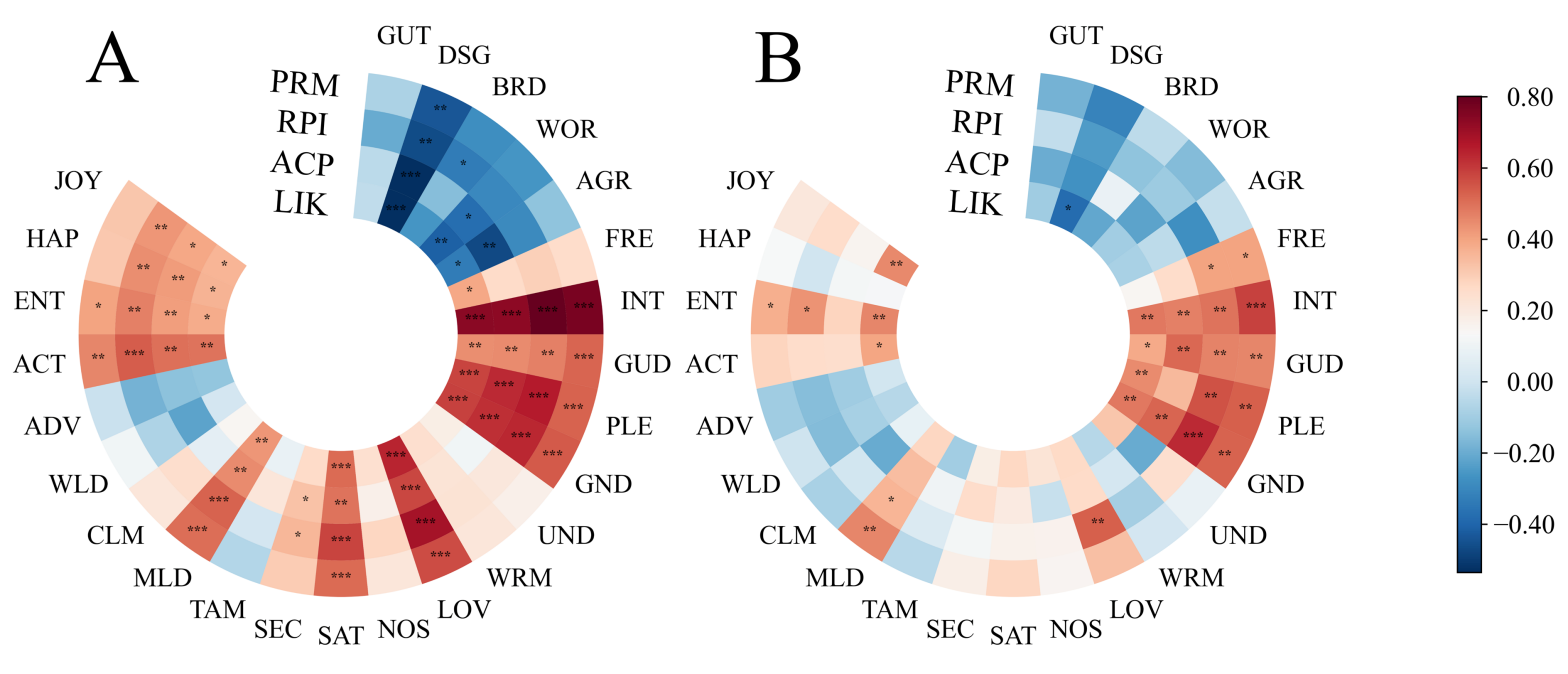
Association Analysis Between Emotion and Preference Terms Based on Repeated-measures Correlation Levels. Note: Statistical significance was corrected using the FDR method (Benjamini–Hochberg, q < 0.05), with * q < 0.05, ** q < 0.01, *** q < 0.001.; (**A**) QR circular heatmap (based on all samples and panelists); (**B**) ETR circular heatmap (based on all samples and panelists).

Repeated-measures correlations between emotional and preference attributes are presented in Figure 5, comprising QR and ETR circular heatmaps (Figure 5A,B; FDR-corrected, q < 0.05). Summary statistics for the distribution of correlation coefficients from both methods are presented in Table 6. In contrast to the sensory–preference and sensory–emotion layers, the two methods showed comparable dispersion (QR: MAD = 0.244; ETR: MAD = 0.211), with a 95% CI [0.77, 1.53] for the MAD ratio (QR/ETR), whose interval encompassed 1.0, indicating no significant difference in dispersion between the two methods [25]. Correlation strength was significantly higher for QR than for ETR (paired Wilcoxon signed-rank test, N = 100, *p* < 0.001). The QR distribution was significantly more negatively skewed than the ETR distribution (QR = −0.530, ETR = −0.005; Bootstrap sign test, *p* < 0.001). Kolmogorov–Smirnov tests further confirmed that the overall distribution shapes differed significantly between the two methods (*p* = 0.036). Among the positive emotional terms with moderate-to-strong correlations (r > 0.5) with more than three preference terms, the following were consistently identified after FDR correction (q < 0.05): QR identified “Loving”, “Interested”, “Pleasant”, “Satisfied” and “Good-natured”; ETR identified only “Good-natured”. The term common to both methods—“Good-natured” (QR: 0.54–0.64; ETR: 0.48–0.63)—was positively associated with preference.

## 4. Discussion

### 4.1. Relationship Between the Two Indices: Consistent but Not Equivalent

The results presented in Figure 2, Figure 3, Figure 4 and Figure 5 indicate that ETR and QR exhibit good consistency. On the one hand, the two methods reached high levels of agreement in both ICC and Spearman correlation and produced an identical overall preference ranking of the samples (R1 > R2 > R3), suggesting that gaze behavior and self-reported ratings share a common evaluative structure. The distribution of a panelist’s attention across the rating scale is therefore not independent of the score ultimately assigned. This finding supports the use of gaze fixation as a process-oriented observational window onto self-reported evaluation.

At the same time, the ETR in the present study captured the allocation of fixation to the scale options during the rating decision rather than direct perception of the product itself. The association between ETR and QR thus arises, in part, because the two indices occupy different stages of a single rating decision chain—attention allocation upstream and score output downstream—which is precisely why they should not be regarded as interchangeable, equivalent measures. The Bland–Altman analysis reinforced this interpretation: although the mean bias approached zero, the 95% limits of agreement were relatively wide, corresponding to roughly 25% of the scale range and implying that substituting one index for the other at the individual level would introduce non-negligible error. Notably, the systematic differences observed in Figure 2 were directional; QR was slightly higher for visual/appearance attributes, whereas ETR was slightly higher for certain emotional attributes. The former may arise because appearance attributes are readily encoded in verbal terms and are therefore more explicitly expressed in self-report, whereas the latter may reflect the lower verbal accessibility of emotional experience, such that attention allocation captures information that verbal report does not fully convey. These directional differences suggest that each index carries information not entirely covered by the other, thereby constituting a complementary rather than redundant relationship.

### 4.2. Differences in Association Strength: Possible Explanations and Boundaries

Across the three association layers—sensory–preference, sensory–emotion, and emotion–preference—the correlation strength of QR was systematically higher than that of ETR (*p* < 0.001); Kolmogorov–Smirnov tests confirmed that the overall distribution shapes differed significantly between the two methods across all three layers (*p* < 0.05), indicating that the two methods consistently produced different distributional forms. However, the dispersion patterns, as measured by MAD, diverged between the layers.

As shown in Figure 3 and Figure 4, and Table 4 and Table 5, the QR coefficients were markedly more dispersed, whereas the ETR coefficients were highly concentrated in the weak-correlation range. This contrast is consistent with the hypotheses of the attentional drift diffusion model (aDDM) [18]. Under this framework, attentional resources are diffusely distributed across attributes early in the decision process, so that the association between any single attribute and the eventual choice is weak, corresponding to the concentrated, low-correlation signature of ETR. Later in the decision, as accumulated evidence approaches the threshold, consumers consciously select readily verbalizable attributes (e.g., gloss, bean aroma) as the basis for preference, corresponding to the high dispersion and stronger correlations of QR [16]. A plausible explanation is that QR represents an output produced after conscious integration, during which semantic associations among attributes (e.g., sweet liking, warmth, good-natured) may be co-activated and thereby inflate the inter-attribute correlations, whereas ETR reflects pre-rating attention allocation that is relatively less affected by semantic integration and consequently yields weaker correlations.

The results in Figure 5 show no significant difference in the dispersion of the correlation coefficients between ETR and QR, indicating that the two methods rank the associations of emotional terms with the preference indices in a consistent manner. Nevertheless, the QR coefficients remained significantly higher than those of ETR. This may be because the ETR adopted here is based solely on the terminal index of “the scale option with the longest fixation duration”, discarding the temporal and trajectory information of gaze and thereby potentially underestimating the true association between gaze behavior and evaluation. In other words, part of the QR > ETR gap may stem from a genuine difference between the two psychological processes, and part may stem from the present ETR index’s incomplete exploitation of the available eye-movement information.

It should be emphasized that the attention to evidence accumulation models commonly invoked to explain preference formation (e.g., aDDM) rely on moment-to-moment fixation sequences to characterize the evidence accumulation process, whereas the ETR used here did not retain such temporal information. The present study is therefore not able to validate or extend these models, and the present account of “attention allocation preceding rating integration” constitutes only a preliminary lead compatible with that theoretical framework. Future studies that incorporate process-oriented indices, such as first fixation direction, the proportion of fixation duration on each option, and the number of revisits, and that record fixations on the product itself (rather than on the rating scale alone) would better clarify the causal and temporal relationships between gaze behavior and evaluative output, and would alone permit a substantive test of the applicability of aDDM in scale rating contexts.

### 4.3. ET-Q Dual-Validation Framework

Conventional check-all-that-apply (CATA) approaches rely on term frequency screening and are therefore susceptible to term accessibility bias [4]. Consumers tend to select terms that are easy to name, yet high-frequency terms are not necessarily genuine preference drivers. The present study proposes that a descriptor should be regarded as a “consensus driver” only when it maintains a stable and relatively strong correlation with preference in both QR (explicit ratings) and ETR (implicit gaze). Applying this criterion, Figure 3 identified warmth and sweet as the core sensory drivers. Among these, warmth was additionally positively associated with positive emotional terms including “Loving” and “Good-natured” (Figure 4), and sweet was additionally positively associated with “Pleasant” (Figure 4), effects that held simultaneously under both methods. Figure 5 identified “Good-natured” as the emotional term jointly endorsed by the two methods. By integrating the dual evidence of explicit semantic judgment and implicit attention allocation, this framework provides clearly defined targets for product optimization.

### 4.4. Contributions and Limitations

Methodologically, this study offers two advances over previous work. Firstly, unlike most previous studies that employed eye tracking and questionnaire measures separately [21], we achieved synchronous acquisition of both measures within the same rating task, enabling a direct and temporally aligned comparison between implicit gaze behavior and explicit self-report. Secondly, while previous studies typically focused on comparing mean differences between methods, we further compared the distributions of correlation coefficients across sensory–emotion–preference layers, providing a more nuanced understanding of how the two measures diverge.

This study also has several limitations. First, ETR was defined as the score corresponding to the longest fixation duration, yet fixation duration may also reflect cognitive difficulty or semantic ambiguity; this definition therefore requires corroboration with first fixation time, fixation counts, or aDDM-based validation. Different eye tracking metrics capture different aspects of processing: first fixation duration reflects initial attention capture, fixation count indicates the salience or informational value of a region, and total dwell time reflects overall engagement with an area of interest [26]. Gaze heatmaps were generated to visualize the spatial distribution of fixations by aggregating fixation positions across participants [26]. Future studies should incorporate a broader range of process-oriented indices, such as first fixation direction and the number of revisits, to more comprehensively characterize gaze behavior during rating decisions. Second, although a sensitivity power analysis indicated that the present sample size (n = 24) was sufficient to detect the core effect size of interest (|r| ≥ 0.5), the sample was small and composed entirely of postgraduate students; and the aim of this study was to test the feasibility of the combined eye tracking and questionnaire approach, with a view to providing technical support and a theoretical basis for subsequent large-scale consumer emotion surveys, and the conclusions are therefore insufficiently representative of consumers at large and require validation in broader populations. Third, only a single beverage—red bean and coix seed water—was examined, so the sample was overly homogeneous; both the sample size and the range of product types should be expanded to establish applicability to complex, multimodal products. Fourth, ETR recorded fixations on the rating scale rather than direct product perception, and the noise arising from this distinction has not yet been systematically evaluated. Finally, the mechanisms underlying the emotion-related associations remain unclear and warrant further exploration using complementary techniques such as electroencephalography, functional near-infrared spectroscopy, or functional magnetic resonance imaging.

## 5. Conclusions

This study employed an area-of-interest (AOI)-based synchronous acquisition paradigm to establish an eye tracking questionnaire (ET-Q) dual-validation framework, addressing the methodological difficulty of aligning implicit and explicit measures on the same temporal scale and information unit in sensory–emotion research. The results indicate that questionnaire ratings (QR) reflect consciously integrated preference judgments, whereas eye tracking-derived ratings (ETR) reflect the allocation of visual attention across the scale options during the rating decision. The two methods showed good reliability and produced an identical preference ranking (R1 > R2 > R3), yet—because they occupy different stages of the rating-decision chain—they cannot be substituted for one another; their relationship is therefore best described as consistent and complementary rather than equivalent.

Using the criterion that a descriptor must correlate stably and significantly with liking under both QR and ETR, the framework identified warmth, smoothness, and sweet as the core sensory drivers, and “Good-natured” as the most robust emotional term, providing cross-validated evidence for product optimization and for the compilation of a standardized sensory lexicon. Because ETR was based solely on the terminal index of the longest-fixated option, and because the sample was small and relatively homogeneous, the present findings are compatible with attention to evidence accumulation models (e.g., aDDM) but are not yet sufficient to test or extend them. Overall, these results should be regarded as exploratory evidence indicating that gaze behavior directed at the response scale can serve as a process-oriented complement to—rather than a replacement for—self-report in sensory–emotion evaluation, and this framework provides additional references for consumer insight research.

## Figures and Tables

**Table 1:** Information about the samples.

Sample ID	Main Ingredients	Color
R1 (347)	Water, adzuki beans, coix seed, erythritol	Yellow, clarity
R2 (634)	Water, adzuki beans, coix seed, erythritol, ascorbic acid, sodium bicarbonate	Yellow, clarity
R3 (932)	Water, adzuki beans, coix seed	Red, clarity

**Table 2:** Sensory lexicon.

Dimension	Descriptors (Abbreviation)
Preference	Liking (LIK), Acceptance (ACP), Re-purchase intention (RPI), Premiumness (PRM)
Visual	Red (RD), Green (GN), Blue (BL), Yellow (YL), Lustre (LST), Clarity (CLY), Uniformity (UNF), Vividness (VVD)
Olfactory	Floral (FLA), Fruity (FRU), Bean aroma (BYN), Grain aroma (GRN), Cooked flavor (CKD), Raw bean flavor (RWB), Rancid (RNC), Burnt (BRT), Off-flavor (OFF)
Taste	Sour (SUR), Sweet (SWT), Salty (SLT), Bitter (BTR)
Tactile	Warmth (WMT), Viscosity (VSC), Graininess (GNS), Silky (SLK), Numbing (NMB), Spicy (SPY), Astringent (AST)

**Table 3:** Emotion lexicon classified by arousal and valence.

Dimension	High Arousal	Low Arousal
High valence	Enthusiastic (ENT), Joyful (JOY), Happy (HAP), Good-natured (GND), Loving (LOV), Free (FRE), Interested (INT), Active (ACT), Adventurous (ADV), Wild (WLD)	Satisfied (SAT), Pleasant (PLE), Good (GUD), Nostalgic (NOS), Mild (MLD), Secure (SEC), Understanding (UND), Tame (TAM), Calm (CLM), Warm (WRM)
Low valence	Aggressive (AGR), Disgusted (DSG)	Guilty (GUT), Worried (WOR), Bored (BRD)

**Table 4:** Table of the distribution of repeated-measures correlation coefficients for Sensory–preference descriptor pairs.

	N	Wilcoxon Test (*p*)	MAD	95% CI of the MAD Ratio	Pearson’s Skewness Coefficient	*p* (Skewness)	K-S Test (*p*)
QR	112	<0.001	0.338	[1.26, 2.01]	−0.028	0.804	0.039
ETR			0.195		0.131		

**Table 5:** Table of the distribution of repeated-measures correlation coefficients for Sensory-Emotion descriptor pairs.

	N	Wilcoxon Test	MAD	95% CI of the MAD Ratio	Pearson’s Skewness Coefficient	*p* (Skewness)	K-S Test (*p*)
QR	700	<0.001	0.184	[1.26, 1.61]	0.196	0.466	<0.001
ETR			0.127		0.258		

**Table 6:** Table of the distribution of repeated-measures correlation coefficients for emotion–preference descriptor pairs.

	N	Wilcoxon Test	MAD	95% CI of the MAD Ratio	Pearson’s Skewness Coefficient	*p* (Skewness)	K-S Test (*p*)
QR	100	<0.001	0.244	[0.77, 1.53]	−0.530	<0.001	0.036
ETR			0.211		−0.005		

## Data Availability

The original contributions presented in this study are included in the article. Further inquiries can be directed to the corresponding authors.
